# Current Approaches to Monitor Macromolecules Directly from the Cerebral Interstitial Fluid

**DOI:** 10.3390/pharmaceutics14051051

**Published:** 2022-05-13

**Authors:** Marie-Laure Custers, Liam Nestor, Dimitri De Bundel, Ann Van Eeckhaut, Ilse Smolders

**Affiliations:** Laboratory of Pharmaceutical Chemistry, Drug Analysis and Drug Information (FASC), Research Group Experimental Pharmacology (EFAR), Center for Neurosciences (C4N), Vrije Universiteit Brussel (VUB), Laarbeeklaan 103, 1090 Brussels, Belgium; marie-laure.custers@vub.be (M.-L.C.); liam.nestor@vub.be (L.N.); dimitri.de.bundel@vub.be (D.D.B.); aveeckha@vub.be (A.V.E.)

**Keywords:** microdialysis, cerebral open flow microperfusion, electrochemical biosensors, macromolecules

## Abstract

Gaining insights into the pharmacokinetic and pharmacodynamic properties of lead compounds is crucial during drug development processes. When it comes to the treatment of brain diseases, collecting information at the site of action is challenging. There are only a few techniques available that allow for the direct sampling from the cerebral interstitial space. This review concerns the applicability of microdialysis and other approaches, such as cerebral open flow microperfusion and electrochemical biosensors, to monitor macromolecules (neuropeptides, proteins, …) in the brain. Microdialysis and cerebral open flow microperfusion can also be used to locally apply molecules at the same time at the site of sampling. Innovations in the field are discussed, together with the pitfalls. Moreover, the ‘nuts and bolts’ of the techniques and the current research gaps are addressed. The implementation of these techniques could help to improve drug development of brain-targeted drugs.

## 1. Introduction

Drug discovery and development processes are a lengthy, costly, uncertain, and thus challenging endeavor [1]. In this respect, designing medicines that specifically target the central nervous system (CNS) adds another layer of complexity. Apart from our still-limited understanding of the CNS, the presence of the blood–brain barrier (BBB) lies at the root of this problem [2].

Since the 1980s, when the first biological drug was approved by the Food and Drug Administration (FDA), the therapeutic landscape has drastically changed [3,4]. Although considerable progress has been made in the treatment of numerous cancers and autoimmune diseases using biologics, patients with neurological diseases cannot yet widely benefit from this revolution in drug development [5,6]. Due to the innate resistance of the BBB to the permeation of large molecules, concentrations of the biologics at the site of action are too low to achieve the desired therapeutic effect [2]. For instance, in June 2021, the FDA approved aducanumab, a first-of-its-kind disease-modifying drug used for the treatment of Alzheimer’s Disease. The FDA approval was groundbreaking in the field of neurology, but highly debated. Moreover, in December 2021, aducanumab was eventually even rejected by the European Medicines Agency because the clinical benefits were ambiguous [5,6,7]. Apart from the rising levels of skepticism toward the validity of the classic amyloid cascade hypothesis, the poor clinical outcome of the biologic can possibly also be attributed to the lack of a brain-targeted drug delivery transport system following systemic administration [8].

Gaining insights into the pharmacokinetic and pharmacodynamic properties of lead compounds is crucial during drug development processes. When it comes to the treatment of brain diseases, collecting information at the site of action is challenging. Cerebrospinal fluid (CSF) concentrations do not necessarily reflect the real concentration in the brain parenchyma. Drug concentrations in the CSF give information regarding drug transport across the choroid plexus, which is the main component of the blood–CSF barrier, but such concentrations do not provide information concerning BBB transport. This misconception hinders progress in the development of drugs targeting the CNS, as explained by Pardridge [9]. Moreover, in recent years, the discovery of the glymphatic system, acting as a clearance pathway in the brain, stirred the debate about brain fluid dynamics even more [10,11,12]. Not only the influx but also the efflux mechanisms importantly impact the brain concentrations of (macro)molecules [13,14].

In fact, there are only a few techniques available that allow for the direct sampling from the cerebral interstitial space and thus provide insight into real concentrations in the brain parenchyma. Such techniques are microdialysis, cerebral open flow microperfusion (cOFM), and biosensors. While these techniques seem promising, they are not (yet) adopted into routine practice. A discussion on their strengths and limitations will be the main focus of this review. The juxtaposition of these three techniques will lead to a more comprehensive overview of recent developments and possibilities in this domain.

Apart from determining exogenous drug concentrations for pharmacokinetic and pharmacodynamic studies, a fundamental part of CNS drug development is the monitoring of endogenous molecules at the site of action to gain insights into the physiology of the brain and its disease processes. Initially, the above-mentioned techniques were developed to monitor neurotransmitters and other small molecules, but over the years, advances in the field have made it possible to monitor macromolecules (neuropeptides, proteins, …) as well. As an example, microdialysis is used to determine cytokine levels [15], but also biomarkers such as amyloid-beta and tau have already been quantified in the cerebral interstitial fluid (ISF) both in mice [16,17,18] and in a clinical setting [19]. Moreover, the use of biosensors for the detection of the biomarker tau has been described as well [20]. A third feature of microdialysis and cOFM is the possibility to locally administer molecules at the site of sampling. This feature can be of interest, as the pharmacological effect (on neurotransmitter levels for example) of the compound under investigation can directly be determined upon its local application at the site of action [21].

Trastuzumab is a prime example of the applicability of these techniques. It is a therapeutic monoclonal antibody that binds to the juxtamembrane region of human epidermal growth factor receptor-2 (HER2) and is successfully used in patients suffering from HER2-overexpressing breast cancer [22]. It is the most used example of personalized medicine. Indeed, because only 30% of breast cancer patients express the HER2 protein, characterization of the molecular profile of patients is required to start treatment [22,23]. Trastuzumab is currently also being evaluated for treatment of patients with brain metastases. Its brain pharmacokinetic profile has been determined in rats and mice using both microdialysis and cOFM [24,25]. Biosensors could be an interesting approach in this regard as well, as they can be used to detect HER2 in cell or tumor lysates [26].

## 2. Technical and Historical Overview

### 2.1. Microdialysis

Microdialysis enables the continuous sampling of endogenous as well as exogenous compounds from the cerebral interstitial space using a probe with a semipermeable membrane. The probe is stereotactically implanted in the brain in the region of interest. A perfusion fluid, mimicking the ISF, often called artificial CSF, is perfused through the probe assembly using a controlled pulse-free syringe pump. At the outlet, the dialysate containing the substances of interest is collected without the need of a pull pump. The dialysate is collected fractionally and subsequently analyzed using a sensitive analytical method of choice. The underlying process for the exchange of substances is based on Fick’s first law of passive diffusion. In addition to the concentration gradient and osmotic pressure, the molecular weight, hydrophobicity, and tertiary structure of the compound, as well as the cut-off and material of the membrane, play key roles in this process [27].

The foundation for the use of microdialysis in its present form, as described above, originates from the early 1960s. The first building blocks were laid by Gaddum [28] by the introduction of a push-pull cannula to collect substances directly from the brain. His work is based on a perfusion technique in subcutaneous tissue described by Fox and Hilton [29], although the cannula was positioned concentrically in the brain tissue to allow for more precise targeting. The development of this in vivo technique evolved from the different attempts to determine neurotransmitters by performing brain dissections followed by post-mortem analyses. Numerous technical problems surrounded these early experiments, such as inaccurate dissections, the validity and correlation of the measurements in post-mortem tissue to the in vivo values, and the fact that only a single measurement of a static moment could be determined [30]. Over the years, it became clear that the in vivo technique had limitations as well that led to numerous adaptations regarding the design of the push-pull cannula. The major bottleneck was the open flow system resulting in tissue damage [31]. To resolve this problem, a cannula was constructed containing a tip covered with a porous semipermeable membrane. This dialysis sac [32], or ‘dialytrode’ as it was called by Delgado et al. [33], was later replaced by a hollow fiber, namely the dialysis membrane [34]. This is the basic principle underlying microdialysis as still referred to nowadays. The major advantage of this innovation is that there is less damage and interference with the brain tissue as exposure of the brain tissue to the perfusate is avoided, making the technique more ‘physiological’ than the push-pull principle [31,34,35].

**Figure 1:**
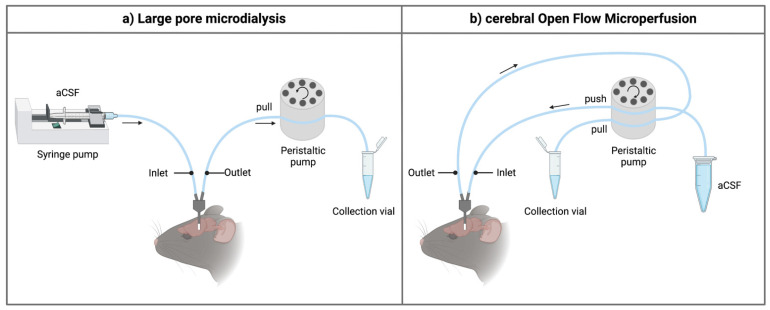
Schematic overview of the inlet and outlet tubings for the sampling techniques. (**a**) When using high-molecular-weight-cut-off microdialysis probes, a push-pull system is required to prevent loss of perfusion fluid through the large pores of the membrane. The setup generally contains a separate controlled pulse-free syringe pump and a peristaltic pull pump (e.g., using an AtmosLM™ or CMA ultra-high cut-off probe). For classical microdialysis, a pull pump is not used. (**b**) For the cerebral open flow microperfusion probe, a peristaltic push-pull microperfusion pump (MPP102 PC, Basi) can be used. Hereby, inflow and outflow can be controlled via the same pump head. Figure created with BioRender.com accessed on 6 April 2022. aCSF: artificial cerebrospinal fluid.

The main component of a microdialysis probe is its semi-permeable membrane. As brain microdialysis was historically applied to gain insight into neurotransmitter levels and other small molecules, cut-off values of the membrane typically ranged from 6 to 40 kDa. Interestingly, the molecular weight cut-off (MWCO) of the microdialysis probe does not reflect the actual pore size of the membrane. It gives information regarding the retention capabilities and thus the sampling efficiency for molecules of a certain size range. For example, a membrane with a 20 kDa MWCO will not allow 80–90% of molecules of that particular size to pass through [36]. Furthermore, there is an exponential decrease in the ability of molecules to pass the semipermeable membrane in relation to an increase in their molecular weight, making classical microdialysis even challenging for sampling molecules with a low molecular weight because of its low recovery rates and low dialysate concentration. Over the years, effort was put in developing probes with a higher cut-off to increase the utility of the technique [15,37,38,39]. At present, probes with cut-off values of 100 kDa–3 MDa are commercially available, allowing the exchange of macromolecules [37,38]. In these kinds of probes, the underlying process for the exchange of substances is primarily based on convection, meaning substances are carried across the membrane pores via bulk-flow together with the solution. Ultrafiltration and, thus, transmembrane pressure (hydrostatic pressure gradient across the membrane) are crucial in this process [39]. To control the fluidic path, thus preventing leakage of the perfusate into the brain parenchyma, a push-pull system is required (Figure 1a). Nevertheless, pressure fluctuations remain a hurdle. Takeda et al. circumvented this problem by introducing a vent hole at the head of the probe assembly (AtmosLM™, Eicom, Green Leaf Scientific, Dublin, Ireland). This vent hole allows for fast equalization of the pressure difference inside the probe with the atmospheric pressure [40].

Samples obtained with classic microdialysis do not require sample clean-up before analysis with liquid chromatography or capillary electrophoresis, as no large molecules are present, because of the low MWCO of the membrane. Because of the high MWCO, analysis of large pore microdialysis samples is generally more challenging [38].

**Figure 2:**
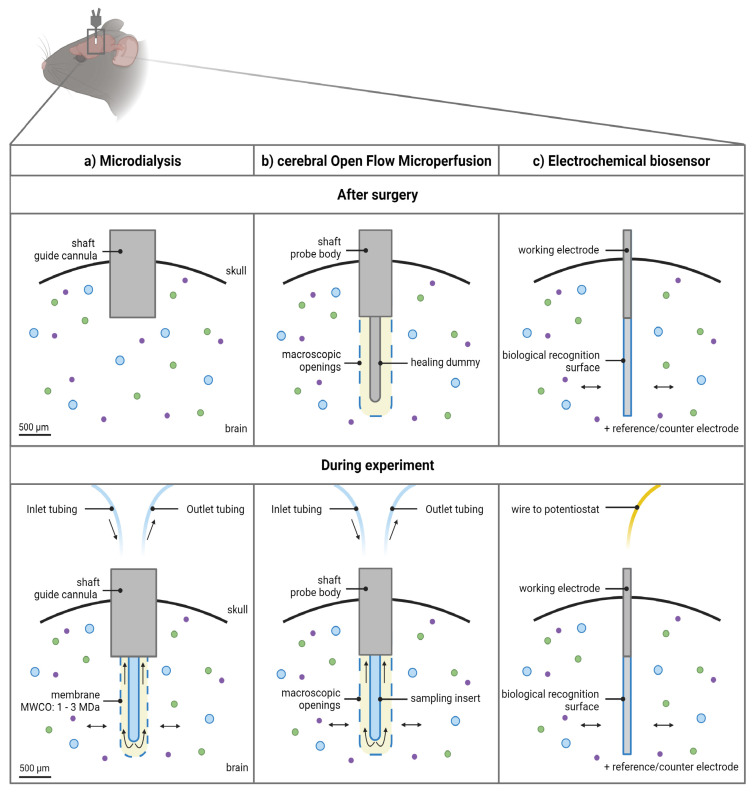
Schematic overview of the 3 approaches to monitor macromolecules directly from the cerebral interstitial fluid. (**a**) The microdialysis probe construct consists of a guide cannula containing a healing dummy implanted in the brain above the region of interest. Before initiating the experiments, the healing dummy is replaced by the probe connected to the tubings. The membrane protrudes beneath the guide cannula. Dimensions of the outer diameter of the shaft of the guide cannula and probe membrane are based on an AtmosLM™ probe. (**b**) The cerebral open flow microperfusion probe body construct/guide itself contains the open exchange area with macroscopic openings and is implanted directly into the brain in the region of interest. Before initiating the experiments, the healing dummy is replaced with the sampling insert connected to the tubings. (**c**) The electrochemical biosensor setup consists of the working electrode (cylinder type is shown), which is implanted in the brain region of interest, while the reference electrode can be implanted in the cortex and the counter electrode can be attached to an anchor screw placed in the skull (not shown). Scale bar indicates 500 µm. Figure created with BioRender.com accessed on 6 April 2022. MWCO: molecular weight cut-off.

Technically, the microdialysis probe construct consists of a guide cannula containing a healing dummy implanted in the brain above the region of interest. Before initiating the sampling experiments, the healing dummy is removed and replaced by the microdialysis probe. The membrane protrudes beneath the guide cannula as illustrated in Figure 2a.

### 2.2. Cerebral Open Flow Microperfusion

Quite soon after its first introduction in the 1960s, the push-pull perfusion technique with its open flow system was put aside. As the use of a membrane has shown its limitations as well (see Section 3), Birngruber et al. went back to the roots and introduced in 2013 an advanced technique referred to as cOFM [41]. The two main features of the patented cOFM probe body design itself are (i) the replacement of the membrane by macroscopic openings and (ii) the biocompatible material (fluorinated ethylene propylene) it consists of [42]. The open structure of the device allows for sampling lipophilic (these tend to adsorb to the microdialysis membrane) and high-molecular-weight substances. The biocompatible material should make it possible to perform chronic sampling experiments as will be discussed in detail in Section 3.1 [43]. Historically, one of the main hurdles to prevent tissue damage by using the push-pull technique was to maintain the probe inlet flow generated by the push pump equal to the probe outlet flow generated by the pull pump [30,31]. This problem was solved by using a pair of high-precision syringe pumps [42]. These were later replaced by a peristaltic microperfusion pump (MPP102 PC, Basi) where inflow and outflow can be controlled via the same pump head (Figure 1b) [25,44].

Technically, the membrane-free cOFM probe body construct itself contains the open exchange area necessary to perform the sampling experiments and is implanted directly into the brain in (not above, as for the microdialysis guide) the region of interest, as illustrated in Figure 2b. It is recommended by the manufacturer to implant the probe body 14 days before initiating the sampling experiments [45]. This recovery period should ensure re-establishment of the BBB (for detailed discussion, see Section 3.1.1) and can be appraised as a third major feature of the probe design [41,42]. A healing dummy prevents tissue growing into the probe during this period. Sampling experiments can be initiated after 14 days by replacement of the healing dummy with a sampling insert [45].

### 2.3. Electrochemical Biosensors

While the two above-mentioned techniques rely on fluid sampling, a third technique, electrochemical biosensors, depends on the recognition of certain molecules directly in the cerebral ISF [46]. The principle of an electrochemical biosensor was first described by Clark and Lyons, in 1962 [47]. A typical biosensor consists of an immobilized, biological recognition element combined with a transducer that converts the biological reaction to a quantifiable signal. The biological recognition element is immobilized on the electrode surface. An electrochemical biosensor uses an electrochemical transducer to convert the signal, which is an electrical current in the case of an amperometric biosensor [48,49,50]. The measured electrical signal is a result of the redox current that is present at the surface of the electrode [51,52]. In this work, the focus is on electrochemical biosensors as this type of biosensor is not only used in fundamental scientific research [53] but also used in clinical practice. For example, the glucose biosensor has been used by diabetic patients for continuous glucose monitoring [50].

The first electrochemical biosensors were developed for the biosensing of molecules by the use of enzymes [50]. Enzymatic biosensors can be divided into three generations. In first-generation biosensors, an enzyme is used as the recognition element and the products used or produced in the enzymatic reaction are in relation to the concentration of the analyte of interest. Second-generation biosensors use electron carriers, while third-generation biosensors do not depend on a mediator but instead use direct electron transfer [51,52]. Enzymatic biosensors still make up the majority of implantable biosensors [54,55]. Apart from enzymes as the biological recognition element, aptamers, antibodies, or antigens can also be used [49,56] to (specifically) recognize the analyte of interest. Nevertheless, interference can be expected by other (small) electroactive compounds. A selective membrane layer/polymer is often used to prevent interference [57,58].

The best examples for the in vivo use of this elegant technique are the glucose biosensor [50] and the assessment of several endogenous small brain molecules such as glutamate [53,59,60] or other molecules [61,62]. While the use of biosensors to establish drug concentrations in the brain is limited, there are examples of biosensors that have been developed for the determination of disease biomarkers [63,64,65] useful for personalized medicine [23,26,66]. Nowadays, biosensors are also being developed for the measurement of large molecules, such as for the detection of tau protein as the biomarker for neurodegenerative diseases [63] or for the detection of hepatitis C virus for diagnostic purposes [67,68,69]. However, the latter are mostly being developed for ex vivo use in biological samples and not yet routinely in vivo. For example, several groups have described the development of a tau protein biosensor, of which an overview can be found in the review by Ameri et al. [20]. The aggregation of tau protein is associated with neurodegeneration and Alzheimer’s disease [17,63]. While the aggregated tau is usually seen in later stages of the disease, it can be of interest to identify soluble tau oligomers during the early stages of the disease. The applicability of biosensors for this purpose is shown in an in vitro setting by Esteves-Villanueva et al. [63]. Another example is the development of an electrochemical biosensor for the determination of the HER2 receptor in cell or tumor lysates, which is the target of the aforementioned trastuzumab monoclonal antibody against cancer and requires characterization of HER2 [26,66].

Typically, in an in vivo setting, a three-electrode system is used: (i) the working electrode (which is the biosensor), (ii) the reference electrode, and (iii) a counter electrode [67,69,70]. Specifically, to monitor extracellular concentrations in the brain tissue, the working electrode(s) is (are) implanted in the brain region of interest (Figure 2c), while the reference electrode can be implanted in the cortex and the counter electrode can be attached to an anchor screw placed in the skull [71].

## 3. Strengths and Limitations

With thousands of publications [27], it can be said that microdialysis is well-established as a technique to gain direct insight into the brain environment. As a consequence, its strengths and limitations are generally well defined, yet controversy still exists in the literature. The three main topics dominating this discussion are the integrity of the BBB, inflammation of the brain tissue surrounding the probe (membrane), and the low recovery rates and associated sticking to the different parts of the setup. cOFM is developed in response to these concerns and attempts to overcome microdialysis’ limitations, although the main principle of the two techniques (and thus its main strengths and limitations) remains the same. On the contrary, while there are some identical advantages and disadvantages between the sampling techniques and electrochemical biosensors, biosensors possess a number of interesting other features (e.g., better spatial/temporal resolution) depending on the aim of the study. Moreover, the use of both techniques can be complementary as they rely on a different principle for the assessment of analyte concentrations. An overview is given in Table 1 and the different topics are explained in detail in the following subsections.

### 3.1. Timeframe to Perform Experiments

When designing a study, the following should be taken into account with regard to the timeframe of the conducted experiments: (i) the recovery period after implantation of the devices in the brain to ensure the integrity of the BBB before initiating an experiment, as well as (ii) the length of the experiment depending on biofouling on the surfaces of the device leading to deviating results.

#### 3.1.1. Blood–Brain Barrier Integrity

All three techniques are commonly referred to as being minimally invasive, although it is clear that the surgical implantation of the probe/electrode has its consequences [72]. Therefore, a recovery and equilibration period should be considered prior to starting the experiments. The literature is not conclusive regarding the integrity of the BBB [41,73,74,75,76,77,78,79,80]. Moreover, manuals available with the different commercialized microdialysis probes do not even provide information about this.

As seen in Figure 2a, the microdialysis probe membrane protrudes beneath the guide cannula. Hence, additional injury is caused when the probe itself is inserted in the brain. Consequently, inserting the microdialysis probe just before the start of the sampling procedure does not provide sufficient recovery time. For example, in Sumbria et al. [73], the integrity of the BBB following microdialysis probe implantation was assessed by determining the extravasation of fluorescent markers, both with a low (sodium fluorescein) and high (Evans Blue) molecular weight, around the probe tract after intravenous administration. The results show an increased extravasation immediately after implantation, but not after 1.5 h or 24 h [73]. In Caljon et al. [74], experiments were started after a 16 h recovery period following microdialysis probe implantation. BBB integrity was assessed using Evans Blue and ^99m^Tc-Sestamibi, revealing no significant extravasation [74], thus all indicating only acute damage immediately after implantation [73,74,77,78,81,82]. Other studies have shown that a biphasic response in BBB permeability occurs, with an increase immediately after probe implantation and a second increase 1–2 days after [75,76,79]. In contrast, Groothuis et al. [75] showed that BBB function is disrupted during at least 28 days after implantation.

cOFM was introduced as a new in vivo technique for measuring substance transport across the intact BBB [41]. The main difference with the microdialysis probe construct is that the cOFM guide cannula contains the open exchange area and is directly implanted in the brain in the region of interest. It was shown that up until 9 days after cOFM probe implantation, the Evans Blue extravasation was still significantly higher than in a negative control, although it decreased to 15%. Therefore, a recovery period of 14 days is recommended by the manufacturer prior to starting cOFM experiments in order to assure BBB integrity [41]. When using a microdialysis probe, the latter is impossible, as is explained in Section 3.1.2. Immediately before cOFM sampling, the healing dummy is replaced by the sampling insert, creating limited new damage according to the manufacturer as the cOFM probe body is implanted in the region of interest and the sampling insert is exactly in-line with the previously implanted guide. The probe is flushed at a high flow (typically 2 min at 5 µL/min) and a 1 h run-in phase is taken into account as an equilibration period [45]. In Custers et al. [44], serotonin and ɣ-aminobutyric acid levels were determined 2 h and 20–24 h after sampling insert replacement to gain insight into possible disturbance of the brain environment, demonstrating that the 1 h run-in phase does not suffice. The BBB is probably disrupted again after insertion of the sampling insert and flushing with the perfusate as it is in direct contact with the brain tissue (because of the absence of a membrane). Most cOFM studies in the literature are performed under anesthesia, which restricts the duration of the sampling experiment itself and makes a longer run-in phase impossible [41,43,45,80,83,84]. In an awake setup, the equilibration period can and should be prolonged [25,44].

Biosensors offer a benefit by the increased spatial resolution and reduction in implantation trauma, as the outer diameter of the implanted device is smaller compared to the sampling probes, as seen in Figure 2 and Table 1. There is no consensus on when cerebral biosensor experiments can be initiated after implantation. The signal can be continuously monitored from the moment just after implantation, although it is important to wait for the baseline to stabilize before initiating an experiment. For example, some groups start measuring minutes after implantation [53,59], while others wait a few hours [71], although the latter strongly depends on the type of electrode that is used and the experimental setup (awake vs. under anesthesia) [85]. Hence, this equilibration period is based on the sensor rather than on brain homeostasis and the integrity of the BBB.

#### 3.1.2. Inflammation

Apart from the recovery/equilibration period before experiment initiation, deciding on the duration of the experimental sampling duration is equally challenging. The key factor limiting the application time and functionality of the techniques is the immunological reaction of the body following implantation of the foreign object and the associated biofouling on its surface [27,72,76,86].

When implanted for too long, the microdialysis membrane becomes clogged (resulting in decreased recovery rates) due to the formation of glial scar tissue and adhesion of molecules onto the membrane. Therefore, it is typically recommended to perform microdialysis experiments for a maximum of 72 h [27,87]. However, chronic microdialysis experiments have also been described in literature [88,89,90,91].

cOFM attempted to overcome this issue by introducing the membrane-free probe and improving the biocompatibility of the probe body using fluorinated ethylene propylene. The study of Birngruber et al. shows no formation of a continuous glial scar up to 30 days after probe implantation in the rat brain [43]. Hence, an advantage of cOFM over microdialysis is the notion that intermittent sampling in a chronic setting should theoretically be more feasible, although abundant literature on this application is not yet available. Commercialized equipment for microdialysis experiments mainly use a guide cannula and probe shaft made of stainless steel (Eicom, Green Leaf Scientific, Dublin, Ireland; CMA Microdialysis AB, Kista, Sweden). A replacement of the metallic material with a guide cannula using a better biocompatible material such as polyether imide/fused silica (Microbiotech/se AB, Stockholm, Sweden) or polyamide/polyurethane (CMA Microdialysis AB) could markedly reduce the immune response as well. There is little reason to believe that fluorinated ethylene propylene can offer better biocompatibility than the other polymers [92], although the presence of the membrane remains a limiting factor. To the extent of our knowledge, no clear comparison exists in the literature between the different available commercial and custom-made microdialysis probe types, although abundant literature is available regarding the inflammatory response. The study of Custers et al. shows that if both a microdialysis probe (AtmosLM™, stainless steel) and cOFM probe are used within their recommended timeframe, the inflammatory response is comparable [44]. Birngruber et al. compared the cOFM probe with a CMA 12 microdialysis probe that consists of a metal-free, biocompatible guide cannula and probe shaft according to the manual (although not specified in the manuscript) [43]. Surprisingly, this study demonstrates a markedly increased astrocytic and microglial reaction for the implanted microdialysis probe 15 days after implantation compared to the cOFM probe [43]. This emphasizes that further research is needed.

Another important factor to consider is the perfusate as there is no consensus on its composition. To minimize an inflammatory response, the physiology of the cerebral ISF should be mimicked, and the solution should be filtered or sterilized. The addition of other components such as bovine serum albumin (BSA) and dextran, which are needed in the context of aspecific adsorption and osmotic pressure, may adversely affect the inflammatory response [44].

In the field of implantable electrochemical biosensors, attention is also paid to avoid biofouling. As is the case with the sampling techniques, the duration of use of these biosensors is also limited as biofouling hampers analyte diffusion toward the biorecognition element, which results in lower sensitivity. Another important point is the inactivation or degradation of the biological recognition element [54,55,93,94,95]. It is well-known that compounds adhere on the surface of the electrode, although efforts are made to limit this. These efforts include the use of naturally occurring or bio-mimicking materials such as chitosan, collagen, and gelatin, but also hydrophilic, superhydrophobic, and drug-eluting materials can be used. Their mechanism of action is, for example, based on the regulation of the host immune response and making the biosensor surface thermodynamically unfavorable for biofouling [55]. For example, in the study of Brown et al. [96], it is shown that exposure of the Nafion^®^-coated platinum sensor to proteins and lipids in an in vitro setting resulted in a decrease in sensitivity up to 24 h, after which levels stabilized. These results were confirmed in vivo up to 8 days after implantation [96]. Generally, chronic in vivo cerebral biosensor experiments are performed for a maximum of 2–3 weeks, although this strongly depends on the sensor used [71,96,97,98].

In summary, regarding microdialysis, it is typically recommended to initiate experiments 16–24 h after probe implantation [73,74], based on BBB integrity, with a maximal duration of 48–72 h [87,99,100], based on the formation of glial scar tissue around the probe that hampers the exchange of molecules [27,76]. Regarding cOFM, experiments can be initiated 14 days after probe implantation [41,80], based on BBB integrity, with a minimal duration of 30 days [43], based on the formation of glial scar tissue [45]. There is no consensus on the timeframe to perform cerebral biosensor experiments. However, the signal can be continuously monitored from the moment upon baseline stabilization after implantation, with a maximum duration of 2–3 weeks, although the latter strongly depends on the type of sensors that are used [71,98].

### 3.2. Recovery Rates

Apart from biofouling, multiple other factors determine the recovery rate of the compound of interest. A first factor determining the recovery rate in microdialysis is the MWCO of the membrane. Furthermore, in addition to lipophilic small molecules, macromolecules such as peptides and proteins tend to adsorb to the membrane. An overview of the modification strategies of the microdialysis membrane surface to reduce aspecific adsorption is given by Van Wanseele et al. [38]. For example, the AN69 membrane offers great potential for reducing aspecific adsorption of peptides [101]. However, next to adsorption of the compound of interest to the probe membrane, sticking can also occur at other parts of the microdialysis setup. Adding BSA to the perfusate to block aspecific binding is commonly used as a main solution together with the use of low-binding tubings. Furthermore, an in vitro adsorption test should be performed prior to in vivo experiments. As for cOFM, the inner lumen of the probe is coated with polytetrafluoroethylene as an additional measure to decrease adsorption [102,103].

The study of Altendorfer-Kroath et al. [83] comparing a 20 kDa microdialysis probe with a cOFM probe, but also the study of Custers et al. [44] comparing a 1 MDa microdialysis probe with a cOFM probe, shows discrepancies between in vitro recovery/adsorption tests compared to the in vivo obtained results. In both studies, the cOFM probe performs better in vitro compared to the microdialysis probe, although this advantage almost completely disappears in an in vivo setting. As problems with ultrafiltration and osmolarity are minimized because of the optimized design of the probe/pump and perfusate, we believe tortuosity of the brain parenchyma can provide a possible answer for this discrepancy [44,104]. To estimate absolute ISF concentrations, for both techniques, in vivo recovery should be determined as well. Typical methods to do so are the no-net-flux approach, the extrapolation-to-zero flow, and the recovery by gain/loss method [27]. Although, in practice, relative concentrations are often used.

To the extent of our knowledge, three studies are available in the literature comparing cOFM with AtmosLM™ to sample macromolecules, namely trastuzumab [25], a brain-penetrating nanobody [44], and tau [18]. Their findings on recovery rates of the macromolecules with the two techniques differ substantially. In the study of Le Prieult et al., 10-fold higher ISF trastuzumab levels were found with cOFM compared to microdialysis when not corrected for in vivo recovery. They also showed that the use of either in vitro or in vivo recovery has a substantial impact on absolute concentrations [25]. In the study of Custers et al., with both techniques, equivalent levels (uncorrected) of a brain-penetrating nanobody were found [44]. The study of Barini et al. shows that ISF sampled by cOFM increased the seeding propensity of tau in the HEK293-tau biosensor cell assay more than ISF sampled through the microdialysis probe. They examined whether this was due to the differential recovery of tau or differential sampling of tau species. Tau levels were significantly higher in AtmosLM™ ISF than in cOFM ISF, although the overall composition of tau fragments was unaffected by the sampling procedure. They hypothesized that the enhanced ability to trigger tau aggregation may require additional ISF components that are only present in cOFM ISF [18]. Because of these divergent results and conclusions, it is clear that there is a need for additional comparative studies with macromolecules of different classes.

Despite in vitro calibrations of the biosensors, the activity of an enzyme is dependent on the environment it is used in. Hence, the activity of the enzyme can be different in vivo versus in vitro and absolute concentrations remain an estimate [54]. In in vivo measurements, baseline currents are measured and changes in this current indicate a change in analyte concentration. Hence, results can be reported as the measured current compared to the baseline current [53,59] but also as concentrations [105]. Both methods of reporting offer a great insight into the change in extracellular concentration of the analyte of interest over time.

### 3.3. Spatiotemporal Resolution

The spatial resolution of a microdialysis and cOFM probe is comparable as the outer diameter is around 500 µm for both and the membrane/open exchange length is typically 1–4 mm depending on the species and target of interest. As a decreased outer probe diameter leads to increased spatial resolution [106], biosensors offer the best spatial resolution out of the three techniques. The outer diameter of a biosensor is typically between 50 and 125 µm, although strongly depending on the type of sensor. Moreover, apart from the cylinder-type biosensors where the biological recognition surface is similar to the membrane/open exchange length, disc-type biosensors have their biological recognition surface located only at the tip of the electrode. These compact disc-type biosensors offer the opportunity to specifically target a brain subregion such as the CA1 region of the hippocampus [54,107].

For microdialysis and cOFM, the limiting factor determining the temporal resolution is the desired sample volume. It depends on the flow rate and associated recovery that, in turn, depends on the subsequent analytical method and is generally minimally in the minutes range [108]. Generally, for cOFM, a flow rate of 0.3–1 μL/min is recommended. Higher flow rates are avoided to prevent tissue damage and analyte depletion in the vicinity of the probe (especially for macromolecules with low concentrations at the site of action) [45]. Lower flow rates are avoided because of the temporal resolution. As for microdialysis, common flow rates are 0.3–2 μL/min [27], because the membrane acts as a protection layer for the brain tissue. On the contrary, biosensors have an optimal temporal resolution in the (milli)seconds range [109,110]. The latter is mainly dependent on the diffusion of the analyte through the membrane layer/polymer [111].

### 3.4. Sample Analysis

Samples obtained with microdialysis or cOFM require a subsequent sensitive analytical method for their analysis. This is not the case for biosensors, which are an analysis method themselves, and the concentration of the analyte is in relation to the obtained current [51].

For the sampling techniques, the information that can be obtained is only as good as the subsequent analytical method. An analytical method requiring a high sample volume, because of a high limit of detection, has great implications on the temporal resolution of the technique. Moreover, because of low recovery rates for some molecules, an ideal analytical method should be sensitive, have a low limit of detection/limit of quantification, and thus require low sample volumes in addition to being validated by the applicable standards [108]. Analytical methods that are mostly used and are extremely well fitted for the analysis of microdialysates are miniaturized liquid chromatography or capillary electrophoresis coupled to mass spectrometry [112,113,114,115], although other detection methods can be suitable. Especially, the use of capillary electrophoresis coupled to mass spectrometry could be interesting because it generally requires very low sample volumes and can thus improve temporal resolution [112,116].

Nevertheless, chronic sampling combined with a high temporal resolution can lead to a great number of samples. Despite the fact that analytical methods are being developed with a short run time, it can become labor-intensive to analyze all these samples. We believe that the use of biosensors has an advantage for chronic sampling, as subsequent sample analysis is not necessary. However, with biosensors, only a limited number of molecules can be monitored simultaneously. In this regard, the use of sampling methods offers a big advantage by enabling the monitoring of a large range of different molecules, for example, in a proteomic screening [117].

Samples collected with large pore microdialysis or cOFM contain, in addition to the analyte of interest, more, other interfering compounds such as proteins, enzymes, and triglycerides compared to classical microdialysis. As a result, these samples cannot be analyzed with the abovementioned methods without doing a sample clean-up [38]. Hence, another possibility is the use of biological assays such as an enzyme-linked immunosorbent assay (ELISA) or single-molecule array (Simoa) where sample pretreatment is a less important factor. Both techniques offer great sensitivity; however, they are expensive and generally require a high sample volume, negatively influencing the temporal resolution [118]. Furthermore, an assay based on LOCI™ (Luminescent Oxygen Channelling Immunoassay) technology can be explored, such as an AlphaLISA™ or AlphaScreen™. The benefit in these kinds of biological assays is that they are quick (require no wash-steps) and very sensitive, allowing detection down to the attomolar (10^−18^) level combined with small sample volumes (<10 µL) [119,120]. However, the small sample volume can impact the validity of the assay.

## 4. Overview of Macromolecules Sampled from the ISF

In Table 2, examples of macromolecules determined in the cerebral ISF using microdialysis or cOFM are given.

Most studies to date that have used microdialysis and cOFM to sample macromolecules concern protein structures. A first class comprises neuropeptides such as hormones [84,137,138], substance P [136], and the neuromedins [114]. From a biochemical point of view, neuropeptides can be situated in the gray zone between small molecules and proteins. With an approximate length of 3–100 amino-acid residues, they are smaller than regular proteins (up to 2000 amino-acid residues) and up to 50 times bigger than the neurotransmitters sampled with classical microdialysis [38,155]. Their quantification in the ISF poses a challenge because of their concentrations in the femto- to picomolar range and sticking behavior to the different parts of the sampling setup. Furthermore, the sampling of larger proteins such as inflammation mediators [40,80,121,122,123,124,125,126,127,128,129,130,131,132,133,134,135] and many markers for neurodegeneration [16,17,18,19,40,146,147,148,149,150] but also growth factors [123,124,125,128,130,131,135] and matrix metalloproteinases [134,135,140,141,142] have been described. Due to their involvement in several neurological disorders, the investigation of their concentration-dependent role at the site of action in the (patho)physiological processes has received attention. Another hot topic is the quantification of nanobodies [44,74] and antibodies [24,25] in the cerebral ISF. Finally, two studies have appeared that sampled liposomes [152], as well as microRNAs [151]. The insight into ISF concentrations of these latter macromolecules is of high interest for the treatment of neurological disorders, thinking, for example, about the potential implementation of monoclonal antibodies as biologics (with or without the use of a shuttling moiety), but RNA therapeutics could also be of interest in this context.

To the extent of our knowledge, electrochemical biosensors are not yet used for in vivo monitoring of macromolecules in the cerebral ISF.

## 5. Innovations in the Field from a Legal Perspective

A research gap exists in the literature regarding the patented innovations for the approaches to monitor macromolecules directly from the cerebral ISF. A search on the term ‘microdialysis’ in the European online database Espacenet, developed by the European Patent Office, yields 3925 hits [156]. The invention of the microdialysis probe as described by Ungerstedt et al. [34] can be seen as the prior art for all the following patents within this field. His invention was patented in 1984 [157] and assigned to CMA Microdialysis AB, one of the major players on the commercial market, as an application in 1993 [158]. Since then, there have been several adaptations made. We believe the current state-of-the-art for sampling macromolecules can be represented by the cOFM probe, invented by Birngruber and Altendorfer-Kroath and patented as a ‘Catheter having a healing dummy’ [41,159,160], and the AtmosLM™ microdialysis probe invented by Nishino et al. [40,161]. However, numerous other inventions have been patented. For example, a patent exists for performing a proteomic study using push-pull microdialysis combined with a MetaQuant probe [162,163]. Stenken and Sellati hold a patent for the sampling of peptides and proteins (e.g., cytokines) in the cerebral ISF using antibody-coated microspheres to enhance recovery [15,164,165]. Regarding biosensors, the patented electrochemical device of Clark in 1959 can be considered the prior art for all innovations within this domain [166]. An overview of all the innovations on the market related to the monitoring of macromolecules in the cerebral ISF would present an excellent guide for researchers within the field.

## 6. Conclusions

The concept of the sampling techniques and biosensors emerged around the same time and they have evolved simultaneously for the past 60 years, depending on the goals of the experiment. Compared to the thousands of microdialysis papers dominating the literature during the past decades, since 2013, a dozen articles using cOFM are published. The latest review comparing cOFM to microdialysis does not present a complete picture, because the open flow system is compared with classical microdialysis probes having a low MWCO [167], although large pore microdialysis is promising as well [37]. Since 2021, three studies were published for the first time comparing a cOFM probe with a large pore microdialysis probe to sample macromolecules [18,25,44]. Regarding electrochemical biosensors, a large body of literature exists as well, although it seems to have had less of a breakthrough in an in vivo setting in the domain of neuroscience compared to microdialysis.

In our opinion, there is no outstanding technique that can replace the others for brain neurochemical monitoring, as they all have their strengths and limitations. The choice of technique should depend on the goal of the study and should consider all factors. In the context of brain-targeted drug delivery, while monitoring, special caution should be taken regarding the integrity of the BBB, and the use of an appropriate control is crucial. Furthermore, statements about ‘real’ concentrations should be considered carefully as it is challenging to determine absolute concentrations in vivo. In conclusion, we strongly believe that the implementation of these techniques leads to a better understanding of the physiology of the brain, is of high importance for pharmacokinetic and pharmacodynamic studies of novel brain-targeted drug candidates, and can thus help to improve the drug discovery and development processes of drugs targeting the CNS.

## Figures and Tables

**Table 1:** Overview of strengths and limitations influencing the possible applications of the three techniques.

	Large Pore Microdialysis	cOFM	Biosensors
**Timeframe**			
- **Start**	After 16–24 h	After 14 days	Upon equilibration
- **Duration**	48–72 h	Up to 30 days	2–3 weeks
**Recovery**	Limited due to membrane	Macroscopic openings	Recognition element
- **Membrane**	Aspecific adsorption	Not applicable	Not applicable
**Spatial resolution**	±500 µm OD		±50–125 µm OD
	Depending on length probe: mm		Cylinder vs. disc
**Temporal resolution**	minutes		(milli)seconds
**Sample analysis**	(Bio-)analytical technique		Not applicable
**Analyte range**	Omics screening possible		Limited
**Others**	Local administration of molecules possible		-

cOFM: cerebral open flow microperfusion, OD: outer diameter.

**Table 2:** Examples of macromolecules sampled from the cerebral interstitial fluid.

	Microdialysis		cOFM	
**Neuropeptides and proteins**	Cytokines	[40,80,121,122,123,124,125,126,127,128,129,130,131,132,133,134,135]	Cytokines	[80]
	TNF-alpha	[40,126]	TNF-alpha	[80]
	Neuromedins	[114]	Leptin	[84]
	Substance P	[136]	Tau	[18]
	Hormones	[137,138,139]	Antibodies	[25]
	Matrix metalloproteinases	[134,135,140,141,142]	Nanobodies	[44]
	Growth factors	[123,124,125,128,130,131,135]		
	S100B	[143,144]		
	Apolipoprotein E	[145]		
	Amyloid beta	[16,19,40,146,147,148]		
	Tau	[17,18,19,146,147,149]		
	Neurofilaments	[19,150]		
	Antibodies	[24,25]		
	Nanobodies	[44,74]		
**Others**	microRNAs	[151]	PEGylated liposomal doxorubicin	[152]
	Prostaglandin E2	[153,154]		

## Data Availability

Not applicable.

## Abbreviations

BBB, Blood–brain barrier; BSA, Bovine serum albumin; CNS, Central nervous system; cOFM, Cerebral open flow microperfusion; CSF, Cerebrospinal fluid; FDA, Food and Drug Administration; HER2, Human epidermal growth factor receptor-2; ISF, Interstitial fluid; MWCO, Molecular weight cut-off; OD, Outer diameter.
